# Methotrexate-related drug reactions on kidneys and liver in rheumatoid arthritis: an analysis of spontaneous reports in EudraVigilance

**DOI:** 10.1186/s13075-025-03551-6

**Published:** 2025-04-05

**Authors:** Kai Khoroshun, Carsten Bantel, Falk Hoffmann, Kathrin Jobski

**Affiliations:** 1 Department of Health Services Research, Carl von Ossietzky Universität Oldenburg, 26111 Oldenburg, Germany; 2 University Department of Anesthesiology, Critical Care, Emergency and Pain Medicine, Klinikum Oldenburg, Oldenburg, Germany

## Abstract

**Objective:**

Methotrexate (MTX), a standard treatment for rheumatoid arthritis (RA), is known for its potential kidney and liver toxicity. Whether concomitant use of analgesics, possibly affecting the same organs, has an impact on the occurrence or course of adverse drug reactions (ADRs) remains unclear.

**Methods:**

We used all spontaneous reports (until 2022) of suspected ADRs associated with MTX in RA patients, from the EudraVigilance database, a spontaneous report system operated by the European Medicines Agency (EMA). We displayed case and treatment characteristics, stratified by the organ affected (kidneys, liver) and the outcome (fatal, non-fatal).

**Results:**

We included a total of 10,319 reports (mean age: 62.3 years, 72.6% female). 365 and 1,082 were related to ADRs involving the kidneys and liver, respectively. Patients with ADRs on the kidneys were older and comedication (e.g. non-steroidal anti-inflammatory drugs (NSAIDs), acetaminophen, metamizole and corticosteroids) was more common than in cases with ADRs on the liver. More patients with kidney- than liver-related ADRs had a fatal outcome (21.1% vs. 5.8%). In fatal cases with ADRs on the kidneys and with ADRs on the liver comedication was more common compared to non-fatal cases.

**Conclusion:**

Liver dysfunction was reported nearly three times more often than renal impairment. However, the kidneys need to be especially watched for, since a fatal outcome was considerably more common in renal failure. More precise and standardized recommendations on renal function tests might be necessary to support physicians in the complex treatment of RA.

**Supplementary Information:**

The online version contains supplementary material available at 10.1186/s13075-025-03551-6.

## Introduction

Methotrexate (MTX) is an antifolate, that has been used for many decades in the European Economic Area (EEA) to treat rheumatoid arthritis (RA) [1, 2]. However, its use is not unproblematic as highlighted in a recent review from the European Medicines Agency (EMA) about its risks [3]. Unaffected of that, MTX is still the standard medication and is used in a low dosage (high dosages are only used in treatment of malignancies), if no contraindications are present [1]. Those include for example certain liver diseases or a reduced renal creatinine clearance [4]. However, limited renal function for example, is very common in RA patients, which can lead to life threatening conditions in combination with MTX use. Further, adverse drug reactions (ADR) like liver and renal toxicity, can occur even in patients without any preexisting conditions [5].

Some analgesics, such as non-steroidal anti-inflammatory drugs (NSAIDs) or acetaminophen, are known to affect the same organs [6, 7]. With pain being a very common symptom in RA, those drugs are often necessary for supportive therapy [7]. Studies have already shown, that concomitant use of NSAIDs and MTX can lead to an increase in blood MTX levels [8]. However, whether this increase has clinical consequences remains unclear. A systematic review from 2010 found only limited drug interactions of MTX and other drugs (e.g. NSAIDs, acetylsalicylic acid (ASA)) and stated, that the clinical significance of these interactions cannot be backed up by extensive clinical observations [9]. Another systematic review from 2011 even called the concurrent use of MTX and NSAIDs safe [10]. Both reviews however made an exception for high dose ASA, which might indeed be problematic [9, 10]. A more recently performed, large register-based study in Denmark on the other hand, found a significantly increased risk of serious ADRs (e.g. liver toxicity and acute renal failure) when MTX was used concomitantly with NSAIDs [11]. Honma et al. used data from the American US Food and Drug Administration’s (FDA) Adverse Event Reporting System (FAERS) to examine drug-drug interaction between low-dose MTX and analgesics. Apart from a few specific NSAIDs, they found no indication of an increase in ADRs with concomitant use of acetaminophen or NSAIDs and MTX. Important to say, some information (e.g. age, sex or comorbidities) that could provide more detailed information regarding the outcome, was not considered [12]. Furthermore, to our knowledge, no large study has yet displayed the fatality of kidney- or liver-related ADRs in RA patients using MTX.

Using data from a European spontaneous reporting system we aimed to display and compare potential characteristics (e.g. MTX use, comedication and outcome) between cases of RA patients using MTX, in which drug reactions affected the kidneys and the liver, respectively.

## Methods

### Study design and data source

We performed a retrospective study with reports from the EudraVigilance database, a spontaneous report system operated by the EMA, with the target to surveil medicines authorized in the EEA [13]. Included were all reports of suspected ADRs associated with MTX until 2022. We submitted a formal request in August 2023 and received a subset of Individual Case Safety Report (ICSR) data elements (cases) coded using the Medical Dictionary for Regulatory Activities (MedDRA) terminology. This standardized, international, hierarchic medical terminology was developed under the guidance of the International Council for Harmonisation of Technical Requirements for Pharmaceuticals for Human Use (ICH) and is used in the registration, documentation and safety monitoring of medical products [14, 15]. We received files providing general information (e.g. the date the report was received), information about the patient (e.g. sex, age, medical history), medication (e.g. indication, start date, end date, route of administration (ROA), dosage), reaction (e.g. reaction type, start date, outcome) and comorbidities linked by individual IDs.

### Identification of cases and variables used

ICSRs with the MedDRA “preferred term” (PT, a distinct descriptor used to represent a single medical concept) “rheumatoid arthritis” as indication and MTX as the interacting or suspected drug were included in the analyses [16]. To determine whether cases had a kidney- or liver-related ADR, the PT of the drug reaction was used. Therefore, a broad MedDRA search was conducted, using standardized MedDRA Queries (SMQs) (for list of used SMQs and most common PTs, see supplementary table S1 and S2). To define the reaction date, we used the start date of the reaction, or if not available, the date the report was first received.

Information about sex and age was used. Age was converted into years if necessary and age groups were created (0–17, 18–39, 40–59, 60–79, 80+). Cases with a missing value for age but the information “fetus”, “neonate”, “infant” or “adolescent”, were included in the age group “0–17”. If the information from the written age group did not match the information from the age in years (e.g. 5 years and elderly), the age in years was considered more reliable.

For comedication, groups for NSAIDs, acetaminophen, metamizole, disease-modifying anti-rheumatic drugs (DMARDs), corticosteroids and folic acid were created, and each case was allocated if possible. If information about the substance was not available, the trade name was used instead. Topically administered NSAIDs and corticosteroids were excluded, using information about the ROA, dose form, substance and brand name. Low dose ASA (not used as pain medication) was identified through information about the dosage or the indication and then excluded. All included NSAIDs were matched to their corresponding Anatomical Therapeutic Chemical (ATC) code (M01A) and grouped on the fifth level. Only DMARDs indicated for the therapy of RA according to the European Alliance of Associations for Rheumatology (EULAR) recommendations [1, 17] were included. All substances were displayed in groups (biological DMARD (bDMARD), conventional synthetic DMARD (csDMARD), targeted synthetic DMARD (tsDMARD)) as well as individually (see supplementary table S3).

MTX use was evaluated for the most recent episode, which was defined by the end date of the last drug administration. If this date was not given in any information of the ICSR, the reaction date, as defined above, was used. The last route of MTX administration was determined using information about the ROA, dose form, dosage and drug name. Because in some cases MTX was last administered via multiple routes (e.g. oral and subcutaneous), more than one last ROA was possible for one ICSR. The groups oral, subcutaneous/subdermal, intramuscular, parenteral, intravenous and other were created. If the duration of last MTX use was given, units were converted into months if necessary. If the duration was missing, it was calculated using the start and the end date. If there was no start date, a duration could not be calculated. If the end date was missing but information about the “action taken with drug” (e.g. “dose not changed”, “dose reduced”, “drug withdrawn”) was available, MTX treatment until the start of the reaction was assumed and the reaction date, as defined above, was used instead [16]. If a case had more than one duration the most recent episode was selected as described above. If there were multiple most recent episodes, the given duration was preferred over the calculated duration and if that information did also not differ, the longer duration was included.

An outcome was considered as fatal, if the outcome was described as “fatal” or the seriousness criteria displayed “results in death”. All other cases (e.g. seriousness criteria “life threatening”, “prolonged hospitalization”, “disabling”, “congenital anomaly” or “other”) were considered non-fatal. For the fatal cases the most common PTs for cause of death were evaluated.

### Statistical analysis

To display demographics, comedication, MTX use and outcome, we calculated descriptive statistics (mean, standard deviation (SD), median, interquartile range (IQR), frequencies and percentages) stratified by the organ affected, by the drug reaction (kidneys, liver) and the outcome (fatal, non-fatal). Additional analyses were conducted for three mutually exclusive groups of cases (i.e., cases with ADRs only affecting the kidneys, cases with ADRs only affecting the liver and cases with ADRs affecting the kidneys and the liver). The five overall most common NSAID drugs were displayed in subgroups. For age, sex, MTX use and outcome, ICSRs with missing or unknown values were excluded, resulting in different denominators.

P-values were derived using t-test (comparison of two groups) or analysis of variance (ANOVA, comparison of three groups) for continuous variables and Fisher’s exact test for dichotomous and categorical variables. Statistical significance was determined at a p-value of less than 0.05.

All statistical analyses were performed using IBM SPSS Statistics for Windows, Version 29 (IBM Corp. Released 2023. Armonk, NY, USA) and SAS, Version 9.4 (SAS Institute Inc., Cary, North Carolina, USA).

## Results

### Characteristics of cases

We received a total of 36,373 MTX-related ICSRs from EudraVigilance, dated from 1950 until 2022. In 10,319 ICSRs, the indication was RA and MTX was the interacting or suspected drug (Table 1). In those ICSRs, 25,893 ADRs spread across 2,965 different PTs were reported. The most common PT was pancytopenia (supplementary table S2). The mainly female patients (72.6%) had a mean age of 62.3 years. Over two thirds (69.9%) of cases had any comedication, the most common being a non-MTX DMARD (43.1%), especially bDMARDs (32.7%), followed by csDMARDs (13.2%). Of the pain medication displayed, NSAIDs were the most used (14.8%), followed by acetaminophen (7.9%) and metamizole (1.2%). In 28.6% of cases, corticosteroids were received and in 23.6% folic acid. MTX was used for a median duration of 13.2 months and administered orally more often than subcutaneously/subdermally (58.9% vs. 27.4%) (for information on further ROA see supplementary table S4). A fatal outcome was reported for 738 (7.5%) of all patients included. For 547 of the 738 fatal cases (74.1%) a cause of death was reported with the most common cause being sepsis (12.3%) followed by multiple organ dysfunction syndrome (11.0%), pneumonia (8.4%) and septic shock (8.4%, supplementary table S5).

**Table 1:** Characteristics of spontaneous reports of MTX-related ADRs by organ affected

Characteristics				Total	ADR on kidneys	ADR on liver
				(*n* = 10319)	(*n* = 365)^1^	(*n* = 1082)^1^
Age, in years				(*n* = 6680)	(*n* = 247)	(*n* = 599)
	Mean (SD)			62.3 (14.2)	65.8 (13.7)	59.6 (13.9)
	Median (Q1-Q3)			64.0 (54.0–72.0)	67.0 (59.0–76.0)	60.0 (51.0–68.0)
Age groups, in years				(*n* = 6686)	(*n* = 247)	(*n* = 599)
	0–17			33 (0.5%)	1 (0.4%)	2 (0.3%)
	18–39			425 (6.4%)	8 (3.2%)	46 (7.7%)
	40–59			2091 (31.3%)	61 (24.7%)	241 (40.2%)
	60–79			3496 (52.3%)	141 (57.1%)	262 (43.7%)
	80+			641 (9.6%)	36 (14.6%)	48 (8.0%)
Sex				(*n* = 10155)	(*n* = 356)	(*n* = 1070)
	Female			7371 (72.6%)	246 (69.1%)	767 (71.7%)
	Male			2784 (27.4%)	110 (30.9%)	303 (28.3%)
Comorbidity				(*n* = 5486)	(*n* = 262)	(*n* = 483)
	Cancerous			548 (10.0%)	29 (11.1%)	42 (8.7%)
Comedication				(*n* = 10319)	(*n* = 365)	(*n* = 1082)
	Any comedication			7218 (69.9%)	302 (82.7%)	694 (64.1%)
	Any NSAID^2^			1527 (14.8%)	86 (23.6%)	171 (15.8%)
		*Most common groups*				
			Propionic acid derivates (M01AE)	522 (5.1%)	40 (11.0%)	64 (5.9%)
			Acetic acid derivates and related substances (M01AB)	494 (4.8%)	32 (8.8%)	54 (5.0%)
			Coxibs (M01AH)	243 (2.4%)	6 (1.6%)	22 (2.0%)
			Oxicam (M01AC)	130 (1.3%)	7 (1.9%)	16 (1.5%)
		*Most common agents*				
			Diclofenac	356 (3.4%)	23 (6.3%)	43 (4.0%)
			Naproxen	216 (2.1%)	15 (4.1%)	19 (1.8%)
			Ibuprofen	207 (2.0%)	16 (4.4%)	30 (2.8%)
			Acetylsalicylic acid	135 (1.3%)	3 (0.8%)	13 (1.2%)
			Celecoxib	122 (1.2%)	4 (1.1%)	12 (1.1%)
	Acetaminophen			813 (7.9%)	50 (13.7%)	96 (8.9%)
	Metamizole			121 (1.2%)	25 (6.8%)	23 (2.1%)
	Non-MTX DMARD			4452 (43.1%)	149 (40.8%)	387 (35.8%)
		bDMARD		3379 (32.7%)	98 (26.8%)	253 (23.4%)
		csDMARD		1361 (13.2%)	67 (18.4%)	160 (14.8%)
		tsDMARD		210 (2.0%)	10 (2.7%)	14 (1.3%)
	Corticosteroids			2947 (28.6%)	139 (38.1%)	266 (24.6%)
	Folic acid			2432 (23.6%)	115 (31.5%)	233 (21.5%)
MTX use						
	Route			(*n* = 7863)	(*n* = 255)	(*n* = 840)
		Oral		4633 (58.9%)	177 (69.4%)	575 (68.5%)
		Subcutaneous/Subdermal		2152 (27.4%)	47 (18.4%)	182 (21.7%)
	Duration in months			(*n* = 6269)	(*n* = 223)	(*n* = 783)
		Mean (SD)		40.3 (59.5)	45.9 (65.0)	30.7 (51.8)
		Median (Q1-Q3)		13.2 (3.0-54.5)	16.2 (3.1–72.0)	9.9 (2.8–34.3)
ADR				(*n* = 10319)	(*n* = 365)	(*n* = 1082)
	On kidneys and liver			67 (0.7%)	67 (18.4%)	67 (6.2%)
Outcome				(*n* = 9906)	(*n* = 365)	(*n* = 1042)
	Fatal			738 (7.5%)	77 (21.1%)	60 (5.8%)

1: For 67 persons we found ADRs affecting kidneys and liver; 2: according to ATC classification

MTX: methotrexate; ADR: adverse drug reaction; NSAID: non-steroidal anti-inflammatory drug; DMARD: disease-modifying anti-rheumatic drug; bDMARD: biological DMARD; csDMARD: conventional synthetic DMARD; tsDMARD: targeted synthetic DMARD

**Fig. 1:**
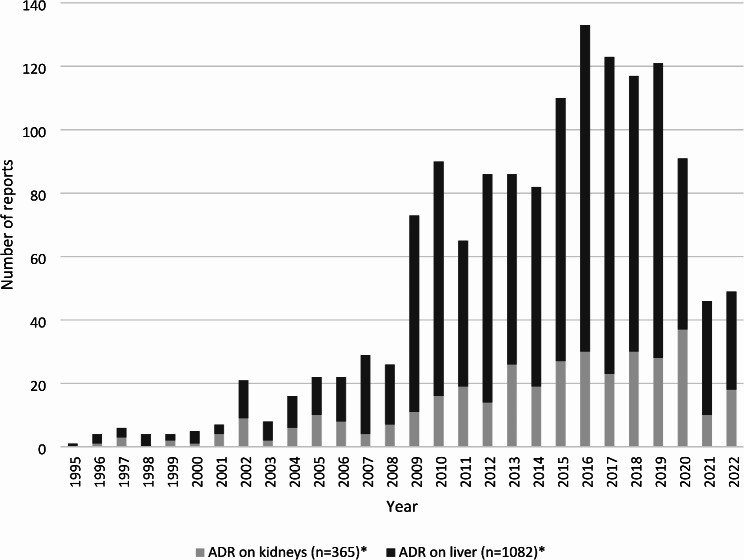
Number of spontaneous reports of MTX-related ADRs by year and organ affected. Note: * = for 67 persons we found ADRs affecting kidneys and liver; MTX: methotrexate; ADR: adverse drug reaction

### Comparison of kidney- and liver-related ADRs

Out of the 10,319 total reports, in 365 cases (3.5%) drug reactions on the kidneys (most often coded PT: acute kidney injury) and in 1,082 cases (10.5%) drug reactions on the liver were reported (most often coded PT: transaminases increased). Most reports for cases with kidney- and liver-related ADRs, were received between 2009 and 2022 (81.3%) (Fig. 1, supplementary table S6). Overall, 67 cases were allocated in both groups, because they had multiple ADRs related to the kidneys and the liver (e.g. renal failure and hepatic function abnormal) or one ADR affecting both organs (e.g. hypoalbuminaemia, hepatorenal failure). Cases with ADRs on the kidneys were older than those with liver-related ADRs (mean age: 65.8 vs. 59.6 years), while the distribution of sex was similar (Table 1). Cases with kidney-related ADRs more often used any comedication (82.7% vs. 64.1%), as well as corticosteroids (38.1% vs. 24.6%). Also, all analgesics were more common in cases with ADRs on the kidneys, with the largest difference in any NSAID (23.6% vs. 15.8%), followed by acetaminophen (13.7% vs. 8.9%) and metamizole (6.8% vs. 2.1%).

Within the group of NSAIDs the largest difference was observed for diclofenac (6.3% vs. 4.0%), naproxen (4.1% vs. 1.8%) and ibuprofen (4.4% vs. 2.8%). Further, the use of any non-MTX DMARD (40.8% vs. 35.8%) was more common in cases with ADRs on the kidneys. While the frequency of oral MTX use was similar (69.4% vs. 68.5%), subcutaneous/subdermal use was less common in cases with ADRs on the kidneys than in those with ADRs on the liver (18.4% vs. 21.7%). We found that in cases with ADRs on the kidneys, MTX was used for a longer time (median duration: 16.2 vs. 9.9 months) and fatal outcomes were more common (21.1% vs. 5.8%) than in cases with ADRs on the liver.

In cases with ADRs on the kidneys a cause of death was reported for 62 of the 77 fatal cases (80.5%), the most common causes being sepsis (21.0%), multiple organ dysfunction syndrome (17.7%) and renal failure (17.7%). In cases with ADRs on the liver a cause of death was reported for 42 of the 60 fatal cases (70.0%), the most common causes being multiple organ dysfunction syndrome (21.4%), hepatic failure (16.7%) and sepsis (14.3%) (supplementary table S5).

When comparing the three mutually exclusive groups of cases (ADR on kidneys only, ADR on liver only, ADR on kidneys and liver) we found substantial differences in their characteristics (supplementary table S7). These could be particularly attributed to the cases with ADRs affecting both the kidneys and the liver which for example displayed the highest proportions of analgesics (NSAIDs (28.4% (kidneys and liver) vs. 22.5% (kidneys) and 15.0% (liver); *p* < 0.001), acetaminophen (23.9% vs. 11.4% and 7.9%; *p* < 0.001) or metamizole (17.9% vs. 4.4% and 1.1%; *p* < 0.001)) and fatal outcomes (31.3% vs. 18.8% and 4.0%; *p* < 0.001).

### Comparison of fatal and non-fatal cases

In 21.1% of the cases with ADRs on the kidneys, the outcome was fatal. Comparing those to the non-fatal cases, the allocation of sex was similar, while fatal cases were slightly older (mean age: 67.5 vs. 65.3 years; *p* = 0.279) (Table 2). Cancerous comorbidities were found somewhat less often in fatal than non-fatal cases (8.6% vs. 11.8%; *p* = 0.638). Almost every comedication displayed was more common in cases with a fatal outcome, with differences for example in corticosteroids (42.9% vs. 36.8%; *p* = 0.356), metamizole (11.7% vs. 5.6%; *p* = 0.074) and acetaminophen (18.2% vs. 12.5%; *p* = 0.196). While the difference in any non-MTX DMARD was small (41.6% vs. 40.6%; *p* = 0.897), it was more apparent in bDMARDs (36.4% vs. 24.3%; *p* = 0.042) and less so in csDMARDs (15.6% vs. 19.1%; *p* = 0.619), with the latter even being more common in non-fatal cases. MTX was used for a significantly shorter period in fatal cases (median duration: 11.0 vs. 16.5 months). An additional ADR on the liver was found in 27.3% of the fatal and 16.0% of the non-fatal cases (*p* = 0.031).

**Table 2:** Comparison of fatal and non-fatal outcomes of spontaneous reports of MTX-related ADRs

Characteristics			ADR on kidneys (*n* = 365)^1^			ADR on liver (*n* = 1042)^1^		
			fatal	non-fatal	*p*-value	fatal	non-fatal	*p*-value
			(*n* = 77)	(*n* = 288)		(*n* = 60)	(*n* = 982)	
Age, in years			(*n* = 57)	(*n* = 190)		(*n* = 42)	(*n* = 549)	
	Mean (SD)		67.5 (12.7)	65.3 (13.9)	0.279	67.5 (16.7)	58.9 (13.5)	< 0.001
	Median (Q1-Q3)		69.0 (60.0–76.0)	65.5 (58.0–76.0)		71.0 (59.0-78.3)	59.0 (50.0–67.0)	
Sex			(*n* = 76)	(*n* = 280)	1.000	(*n* = 58)	(*n* = 972)	0.881
	Female		53 (69.7%)	193 (68.9%)		41 (70.7%)	696 (71.6%)	
	Male		23 (30.3%)	87 (31.1%)		17 (29.3%)	276 (28.4%)	
Comorbidity			(*n* = 58)	(*n* = 204)		(*n* = 43)	(*n* = 427)	
	Cancerous		5 (8.6%)	24 (11.8%)	0.638	7 (16.3%)	34 (8.0%)	0.084
Comedication			(*n* = 77)	(*n* = 288)		(*n* = 60)	(*n* = 982)	
	Any comedication		67 (87.0%)	235 (81.6%)	0.311	52 (86.7%)	613 (62.4%)	< 0.001
	Any NSAID^2^		19 (24.7%)	67 (23.3%)	0.765	12 (20.0%)	154 (15.7%)	0.365
	Acetaminophen		14 (18.2%)	36 (12.5%)	0.196	13 (21.7%)	83 (8.5%)	0.002
	Metamizole		9 (11.7%)	16 (5.6%)	0.074	6 (10.0%)	17 (1.7%)	0.001
	Non-MTX DMARD		32 (41.6%)	117 (40.6%)	0.897	25 (41.7%)	343 (34.9%)	0.330
		bDMARD	28 (36.4%)	70 (24.3%)	0.042	17 (28.3%)	223 (22.7%)	0.343
		csDMARD	12 (15.6%)	55 (19.1%)	0.619	11 (18.3%)	141 (14.4%)	0.449
		tsDMARD	1 (1.3%)	8 (3.1%)	0.695	1 (1.7%)	13 (1.3%)	0.566
	Corticosteroids		33 (42.9%)	106 (36.8%)	0.356	23 (38.3%)	227 (23.1%)	0.012
	Folic acid		25 (32.5%)	90 (31.3%)	0.890	18 (30.0%)	203 (20.7%)	0.103
MTX use								
	Route		(*n* = 58)	(*n* = 197)		(*n* = 51)	(*n* = 759)	
		Oral	42 (72.4%)	135 (68.5%)	0.629	38 (74.5%)	525 (69.2%)	0.530
		Subcutan/Subdermal	12 (20.7%)	35 (17.8%)	0.700	10 (19.6%)	166 (21.9%)	0.861
	Duration in months		(*n* = 45)	(*n* = 178)		(*n* = 34)	(*n* = 725)	
		Mean (SD)	31.8 (44.5)	49.5 (68.8)	0.038	43.4 (60.9)	30.4 (52.0)	0.159
		Median (Q1-Q3)	11.0 (1.7–50.8)	16.5 (3.6–74.5)		18.7 (2.0-60.5)	9.7 (2.7–34.1)	
ADR			(*n* = 77)	(*n* = 288)		(*n* = 60)	(*n* = 982)	
	On kidneys and liver		21 (27.3%)	46 (16.0%)	0.031	21 (35.0%)	46 (4.7%)	< 0.001

1: For 67 persons we found ADRs affecting kidneys and liver; 2: according to ATC classification

MTX: methotrexate; ADR: adverse drug reaction; NSAID: non-steroidal anti-inflammatory drug; DMARD: disease-modifying anti-rheumatic drug; bDMARD: biological DMARD; csDMARD: conventional synthetic DMARD; tsDMARD: targeted synthetic DMARD

A fatal outcome was reported in 5.8% of cases with ADRs on the liver. Compared to non-fatal cases, female sex was similar, while fatal cases were significantly older (mean age: 67.5 vs. 58.9 years; *p* < 0.001) and a cancerous comorbidity was more common (16.3% vs. 8.0%; *p* = 0.084). Also, every comedication displayed was more common in the fatal liver cases, with significant differences in corticosteroids (38.3% vs. 23.1%; *p* = 0.012), acetaminophen (21.7% vs. 8.5%; *p* = 0.002), metamizole (10.0% vs. 1.7%; *p* = 0.001) and the use of any comedication (86.7% vs. 62.4%; p = < 0.001). Contrary to the kidney cases, MTX was used for a longer period in liver cases with a fatal outcome (median duration: 18.7 vs. 9.7 months). An additional ADR on the kidneys was found in 35.0% of the fatal and 4.7% of the non-fatal cases (*p* < 0.001).

## Discussion

In 10,319 spontaneous reports of RA patients using MTX, we found 365 cases of kidney-related and 1,082 cases of liver-related drug reactions. Patients with ADRs on the kidneys were older, used MTX for a longer time and the majority of the comedication displayed was more common than in patients with ADRs on the liver. A fatal outcome was almost three times more common in cases with ADRs on the kidneys. Patients with liver-related ADRs less often used comedication. ADRs affecting both organs were only reported in 67 cases, however, those displayed the highest proportion of fatal outcomes.

### Frequency of kidney- and liver-related ADRs

We found over three times as many cases with liver-related than with kidney-related ADRs. In their systematic review, Wang et al. reported a frequency of 70% for hepatotoxicity and found elevated liver enzymes in 14–35% of RA patients treated with MTX. Nephrotoxicity was described as “quite common”, without presenting specific numbers [5]. While the real proportion of kidney- to liver-related ADRs remains unclear, this study shows that reports of ADRs on the liver are more common than reported ADRs involving the kidneys. A cross-sectional study of 150 RA patients treated with MTX, found impaired renal function in 6% and indicators for reduced liver function in 31% of the cases [18]. An analysis of spontaneous reports from FAERS found a lower proportion of hepatotoxicity in RA cases with MTX-related ADRs than we did (3.1% vs. 10.5%) [19].

One reason why reports on kidney-related ADRs were comparatively rare in our study, could be that early impairment of renal function has no clinical symptoms and can only be detected through blood tests [5]. Consequently, mild or temporary renal impairment related to MTX might go unnoticed and unreported. Unfortunately, there is no standard recommendation on how frequently renal function should be tested. While EULAR recommendations provide no information on this topic [1], some German summaries of product characteristics (SmPC) do, however, with inconsistent or unprecise testing intervals even for the same drug (MTX) [20, 21]. Additionally, increased awareness due to concerns of hepatotoxicity in patients using DMARDs and investigations by the EMA in the early 2000s [22], may have led to closer monitoring of liver function, potentially resulting in more frequent and earlier reports of liver-related ADRs.

### Liver

Overall, comedication was less common in cases with ADRs on the liver. This also applied to analgesics when compared to cases with kidney-related ADRs. Honma et al., who used FAERS to examine drug-drug interaction between low-dose MTX and analgesics, found no positive signals for concomitant acetaminophen and MTX use, indicating it does not increase hepatotoxicity risk [12]. The comparatively low frequency of acetaminophen use in our cases with ADRs on the liver may be due to its avoidance in patients with a known risk for liver-related ADRs (confounding by indication) [5].

The small differences in comedication suggest they may not contribute largely to the appearance of ADRs on the liver. However, some drugs can increase hepatotoxicity risk in patients with altered liver function [23], which could affect outcomes in those with liver-related comorbidities. This is supported by the higher proportion of comedication in fatal versus non-fatal cases with ADRs on the liver. This was also the case for metamizole. While agranulocytosis is one of its commonly known ADRs, there have been recent reports of drug-induced liver injury (DILI). Sebode et al. analyzed the drug causing DILI in 154 patients. In their clinical practice they found metamizole to be the second most common reason for DILI. Some cases even developed acute liver failure needing transplantations [24]. Consequently, in Germany where metamizole is commonly used, a warning about DILI risks has been published [25]. However, this study cannot determine whether the higher metamizole use in fatal cases is due to DILI, agranulocytosis or other factors.

On average, cases with ADRs on the liver were younger and used MTX for a shorter time, in most cases for less than one year. The difference in age could be due to the fact, that biological age seems to have less effect on liver compared to kidney function [26]. Concerning the time MTX was used, it is uncertain whether the ADRs have an earlier onset or are just detected sooner, as described before. Either way, our findings are congruent with studies on liver enzyme elevation in patients using MTX, for they found a mean time between therapy start and first elevation of approximately 18 to 22 months with also over 50% occurring within the first year [27, 28].

We found fatal cases to be more than eight years older on average and to use MTX for a longer time than non-fatal cases. This is not surprising, since the previously described temporary elevation of liver enzymes occurs early but is usually self-limiting. More severe ADRs on the liver, such as hepatic fibrosis or cirrhosis, develop later in treatment and can be life-threatening. Those however, do not occur often, which again agrees with our low number of overall fatal outcomes in cases with liver-related ADRs [29]. Even though ADRs on the liver seem to appear rather early and to younger people, fatal cases have a later onset and affect older people. Therefore, older patients should be monitored closely, not only at the beginning of the therapy, to avoid fatal outcomes.

### Kidneys

Patients with a reported ADR on the kidneys displayed the highest frequency of comedication. In agreement with Honma et al. [12], we found acetaminophen to be the most common analgesic used concomitantly with MTX. Further, diclofenac and ibuprofen were among the five most common agents in cases with ADRs on the kidneys in both studies. Differences with respect to other analgesics might be attributed to the fact that we only included reports from the EEA and, in contrast to Honma et al., excluded low dose ASA in our study.

We found that NSAIDs were more commonly used in cases with ADRs on the kidneys, which seems plausible since they are known to be nephrotoxic and renal impairment can occur as an adverse event [7]. Additionally, a concomitant use can lead to elevated blood MTX levels [8, 30], potentially worsening the nephrotoxic effects of MTX [5]. In support of this, Svanström et al. found a significantly increased risk of acute renal failure with concomitant use of MTX and NSAIDs [11]. Honma et al. on the other hand, only described an increased risk of renal failure for a few NSAIDs when used concomitant with MTX compared to the sole use of those NSAIDs [12]. Therefore, the true clinical effect remains uncertain. In our study, coxibs were the only NSAID less common in cases with ADRs on the kidneys, suggesting a lesser impact on renal function. In two randomized controlled studies Schwartz et al. found no significant effect of etoricoxib on oral MTX, regarding the risk of ADRs, at dosages up to 90 mg and incongruent results for higher dosages [31]. However, like other NSAIDs (e.g. diclofenac), coxibs can affect the cardiovascular system [32], complicating RA treatment, since RA itself is also a risk factor for cardiovascular events [7]. Therefore, caution should be taken when considering NSAID treatment in patients with RA.

Other commonly used medications are corticosteroids, which we found in more than one third of cases with ADRs on the kidneys. According to EULAR recommendations, corticosteroids should only be used as a bridging therapy in the beginning of the treatment and for no more than six months. However, there are some exceptions, for example if the treatment strategy needs to be adjusted by adding or replacing a DMARD or if flares occur [1, 33]. The large number of cases with concomitant corticosteroid use in our study could therefore indicate many cases in early treatment phases. However, the comparatively high median duration of MTX use (16.2 months), makes this unlikely. It could rather suggest difficult treatment or many flares in these cases, indicating a more severe disease state and making prolonged corticosteroid use necessary. This is supported by the findings of Caplan et al., who showed that corticosteroid use is associated with the severity of RA [34] and our findings of more corticosteroid use in fatal, compared to non-fatal cases.

With over 20% of the cases, fatal outcomes were reported almost four times more often in patients with ADRs on the kidneys than in those with ADRs on the liver. Among cases with kidney-related ADRs, we also found kidney- and liver-related comorbidities to be more common in those with fatal outcomes (data not shown), suggesting that these preexisting conditions might influence the outcome. Since comorbidities and ADRs might not be unambiguously reported, our data does not allow for a definitive conclusion regarding this aspect. However, studies have shown that previous health condition (e.g. chronic kidney or liver diseases, prior acute kidney injury) is a predictor of poor prognosis and potentially leads to a higher mortality in patients with kidney impairment [35]. Therefore, to reduce the large number of fatal outcomes, closer monitoring (e.g. standardized blood tests) might be necessary, especially during the first year of treatment, where more than half of the fatal cases occurred.

### Strengths and limitations

The major strength of this study is the large case number, broad time span and international scope, allowing for comprehensive findings. However, as important as data from spontaneous reporting systems are for pharmacovigilance, it also has certain limitations. For one, underreporting is a known limitation for this kind of data [36], gaining importance the longer drugs have been on the market and the more well known an ADR is [37]. Conversely, stimulated reporting can occur when medical or mass media attention is drawn to a certain drug or ADR [38], as seen with MTX over the years [3]. However, these factors would equally affect the report of kidney- and liver-related ADRs and therefore are unlikely to have an influence on the reports. The previously mentioned latest discussion about liver toxicity of MTX did most likely not influence the report number yet, for it is too recent. Our data provided in Fig. 1 supports these assumptions. The distribution of kidney- and liver-related cases and therefore our results could have been influenced by our search strategy in MedDRA. We included 308 PTs for kidney-related and 348 PTs for liver-related ADRs (for further information see supplementary table S1) and therefore a number at least equal to comparable studies [12, 19]. A different strategy could lead to a larger number of included PTs, while also increasing the risk of accidentally including PTs that are unsuitable for the actual aim of the study. Further, the quality of spontaneous reports is another limitation [39], addressing both incomplete and inconclusive data. We therefore used many different variables including free texts to gain as much information as possible (e.g. regarding the ROA for MTX, the variables dose form, dosage and drug name were included). To address inconclusive data, we performed extensive plausibility checks. The aspect of inconclusive data particularly applied to the variables concerning MTX dosage which precluded further analyses. However, since we included only patients with RA as an indication for MTX, we expect the vast majority to have received low-dose MTX. Lastly, due to the nature of the data, it is not possible to calculate a prevalence, incidence or risk, since the total number of MTX users is not accessible.

## Conclusion

Among all reported ADRs, liver dysfunction was nearly three times more often than renal impairment. However, the kidneys are the organs that need to be especially watched for, since a fatal outcome was reported considerably more often. Because drug management in patients with RA using MTX is a complex matter, precise and standardized recommendations on when and how frequently renal function needs to be tested to detect early signs of renal impairment might be helpful to prevent fatal outcomes.

## Electronic supplementary material

Below is the link to the electronic supplementary material.

Supplementary Material 1

## Data Availability

The dataset supporting the conclusion of this article is available from EMA. Restrictions apply to the availability of these data, which were used under license for this study. Data is available from the authors with the permission of EMA.
