# Supplementary material for: Methotrexate-related drug reactions on kidneys and liver in rheumatoid arthritis: an analysis of spontaneous reports in EudraVigilance
Source: Arthritis Res Ther. 2025 Apr 5;27:80. doi: 10.1186/s13075-025-03551-6 (PMC11972469; doi:10.1186/s13075-025-03551-6)
Supplement: Supplementary file 1 — Supplementary Material 1 [file 13075_2025_3551_MOESM1_ESM.docx]

| **Table S1** – Complete list of SMQs used for definition of cases and comorbidities | | | | | | | | |
| --- | --- | --- | --- | --- | --- | --- | --- | --- |
| **Group** | | **SMQ** | | | | | **Total number of PTs** | **Number of narrow PTs** |
| ADR on kidneys | |  |  |  |  |  |  |  |
|  |  | Acute renal failure (20000003) | | | | | 52 | 19 |
|  |  | Chronic kidney diseases (20000213) | | | | | 202 | 42 |
|  |  | Tubulointestinal diseases (20000221) | | | | | 54 | 25 |
| ADR on liver | |  |  |  |  |  |  |  |
|  |  | Drug related hepatic disorders – comprehensive search (20000006) | | | | | 348 | 283 |
|  |  |  | Cholestasis and jaundice of hepatic origin (20000009) | | | | 18 | 16 |
|  |  |  | Drug related hepatic disorders – severe events only (20000007) | | | | 170 | 145 |
|  |  |  |  | Hepatic failure, fibrosis and cirrhosis and other liver damage-related conditions (20000013) | | | 112 | 91 |
|  |  |  |  | Hepatitis, non-infectious (20000010) | | | 25 | 22 |
|  |  |  |  | Liver neoplasms, benign (incl cysts and polyps) (20000012) | | | 12 | 12 |
|  |  |  |  | Liver neoplasms, malignant and unspecified (20000011) | | | 21 | 20 |
|  |  |  |  |  | Liver malignant tumours (20000208) | | 19 | 18 |
|  |  |  |  |  | Liver tumours of unspecified malignancy (20000209) | | 2 | 2 |
|  |  |  | Liver related investigations, signs and symptoms (20000008) | | | | 115 | 79 |
|  |  |  | Liver-related coagulation and bleeding disturbances (20000015) | | | | 45 | 43 |
| SMQ: standardized MedDRA (Medical Dictionary for Regulatory Activities) Queries; PT: preferred term; ADR: adverse drug reaction | | | | | | | | |
| Note: Some PTs are listed in multiple SMQs | | | | | | | | |

| **Table S2** – 10 most common PTs for ADRs in each case group | | |
| --- | --- | --- |
| **Name of PT** | | **Frequency** |
| Total (2965 different PTs) | | (n=25893)^3^ |
|  | Pancytopenia | 779 (3.0%) |
|  | Nausea | 740 (2.9%) |
|  | Drug ineffective | 394 (1.5%) |
|  | Pneumonia | 362 (1.4%) |
|  | Vomiting | 354 (1.4%) |
|  | Dyspnea | 321 (1.2%) |
|  | Interstitial lung disease | 314 (1.2%) |
|  | Pyrexia | 295 (1.1%) |
|  | Thrombocytopenia | 279 (1.1%) |
|  | Leukopenia | 267 (1.0%) |
| Kidney (47 different PTs)^1^ | | (n=442)^4^ |
|  | Acute kidney injury | 102 (23.1%) |
|  | Renal failure | 78 (17.6%) |
|  | Renal impairment | 43 (9.7%) |
|  | Blood creatinine increased | 28 (6.3%) |
|  | Pericarditis | 24 (5.4%) |
|  | Hematuria | 21 (4.8%) |
|  | Encephalopathy | 15 (3.4%) |
|  | Hyponatremia | 14 (3.2%) |
|  | Chronic kidney disease | 11 (2.5%) |
|  | Hypoalbuminemia | 11 (2.5%) |
| Liver (78 different PTs)^2^ | | (n=1463)^5^ |
|  | Transaminases increased | 177 (12.1%) |
|  | Alanine aminotransferase increased | 154 (10.5%) |
|  | Hepatic function abnormal | 96 (6.6%) |
|  | Aspartate aminotransferase increased | 95 (6.5%) |
|  | Hepatic enzyme increased | 91 (6.2%) |
|  | Hypertransaminasemia | 80 (5.5%) |
|  | Liver function test abnormal | 68 (4.6%) |
|  | Gammaglutamyltranferase increased | 52 (3.6%) |
|  | Hepatotoxicity | 45 (3.1%) |
|  | Hepatitis | 44 (3.0%) |
| 1: additional 208 PTs included but reported in none of the cases; 2: additional 252 PTs included but reported in none of the cases; 3: spread across 10319 reports; 4: spread across 365 reports; 5: spread across 1082 reports | | |
| PT: preferred Term; ADR: adverse drug reaction | | |

| **Table S3** – Frequency of specific DMARDs by organ affected | | | | |
| --- | --- | --- | --- | --- |
|  | | **Total** | **ADR on kidneys** | **ADR on liver** |
|  |  | **(n=10319)** | **(n=365)^1^** | **(n=1082)^1^** |
| bDMARD | |  |  |  |
|  | Etanercept | 931 (9.0%) | 25 (6.8%) | 47 (4.3%) |
|  | Adalimumab | 885 (8.6%) | 34 (9.3%) | 72 (6.7%) |
|  | Infliximab | 568 (5.5%) | 21 (5.8%) | 36 (3.3%) |
|  | Tocilizumab | 443 (4.3%) | 13 (3.6%) | 58 (5.4%) |
|  | Abatacept | 348 (3.4%) | 8 (2.2%) | 26 (2.4%) |
|  | Rituximab | 345 (3.3%) | 15 (4.1%) | 28 (2.6%) |
|  | Certolizumab-Pegol | 145 (1.4%) | 5 (1.4%) | 15 (1.4%) |
|  | Golimumab | 129 (1.3%) | 5 (1.4%) | 11 (1.0%) |
|  | Anakinra | 31 (0.3%) | 3 (0.8%) | 1 (0.1%) |
|  | Sarilumab | 20 (0.2%) | 3 (0.8%) | 6 (0.6%) |
| csDMARD | |  |  |  |
|  | Leflunomide | 551 (5.3%) | 28 (7.7%) | 81 (7.5%) |
|  | Chloroquine | 538 (5.2%) | 22 (6.0%) | 51 (4.7%) |
|  | Sulfasalazine | 430 (4.2%) | 17 (4.7%) | 49 (4.5%) |
|  | Ciclosporin | 85 (0.8%) | 11 (3.0%) | 15 (1.4%) |
|  | Gold | 43 (0.4%) | 1 (0.3%) | 4 (0.4%) |
|  | Azathioprine | 43 (0.4%) | 2 (0.5%) | 7 (0.6%) |
|  | Cyclophosphamide | 17 (0.2%) | 1 (0.3%) | 1 (0.1%) |
| tsDMARD | |  |  |  |
|  | Tofacitinib | 121 (1.2%) | 7 (1.9%) | 9 (0.8%) |
|  | Baricitinib | 71 (0.7%) | 3 (0.8%) | 3 (0.3%) |
|  | Upadacitinib | 22 (0.2%) | 2 (0.5%) | 2 (0.2%) |
|  | Filgotinib | 1 (0.0%) | 0 (0.0%) | 0 (0.0%) |
| 1: For 67 persons we found ADRs affecting kidneys and liver | | | | |
| DMARD: disease-modifying anti-rheumatic drug; ADR: adverse drug reaction; bDMARD: biological DMARD; csDMARD: conventional synthetic DMARD; tsDMARD: targeted synthetic DMARD | | | | |

| **Table S4** - Route of MTX-administration by organ affected | | | |
| --- | --- | --- | --- |
|  | **Total** | **ADR on kidneys** | **ADR on liver** |
|  | **(n=7863)** | **(n=255)^1^** | **(n=840)^1^** |
| Oral | 4633 (58.9%) | 177 (69.4%) | 575 (68.5%) |
| Subcutaneous/Subdermal | 2152 (27.4%) | 47 (18.4%) | 182 (21.7%) |
| Intramuscular | 547 (7.0%) | 20 (7.8%) | 64 (7.6%) |
| Parenteral (not further specified) | 458 (5.8%) | 9 (3.5%) | 20 (2.4%) |
| Intravenous | 117 (1.5%) | 3 (1.2%) | 11 (1.3%) |
| Other^2^ | 35 (0.4%) | 1 (0.4%) | 2 (0.2%) |
| 1: For 47 persons we found ADRs affecting kidneys and liver; 2: e.g. retrobulbar, intraarticular, ophthalmic | | | |
| MTX: methotrexate; ADR: adverse drug reaction | | | |

| **Table S5** - 5 most common causes of death in each case group | | |
| --- | --- | --- |
| **Cause of death** | | **Frequency** |
| Total | | (n=547)^1^ |
|  | Sepsis | 67 (12.3%) |
|  | Multiple organ dysfunction syndrome | 60 (11.0%) |
|  | Pneumonia | 46 (8.4%) |
|  | Septic shock | 46 (8.4%) |
|  | Pancytopenia | 36 (6.6%) |
| Cases with ADR on kidneys | | (n=62)^2^ |
|  | Sepsis | 13 (21.0%) |
|  | Multiple organ dysfunction syndrome | 11 (17.7%) |
|  | Renal failure | 11 (17.7%) |
|  | Septic shock | 7 (11.3%) |
|  | Pneumonia | 6 (9.7%) |
| Cases with ADR on liver | | (n=42)^3^ |
|  | Multiple organ dysfunction syndrome | 9 (21.4%) |
|  | Hepatic failure | 7 (16.7%) |
|  | Sepsis | 6 (14.3%) |
|  | Renal failure | 5 (11.9%) |
|  | Death | 4 (9.5%) |
| 1: in 191 fatal cases no cause of death was given; 2: in 15 fatal cases no cause of death was given; 3: in 18 fatal cases no cause of death was given | | |
| ADR: adverse drug reaction  Note: for some cases multiple causes of death were reported | | |

| **Table S6** – Number of spontaneous reports of MTX-related ADRs by organ affected per year | | |
| --- | --- | --- |
| **Year** | **ADR on kidneys** | **ADR on liver** |
|  | **(n=365)^1^** | **(n=1082)^1^** |
| 1995 | 0 | 1 |
| 1996 | 1 | 3 |
| 1997 | 3 | 3 |
| 1998 | 0 | 4 |
| 1999 | 2 | 2 |
| 2000 | 1 | 4 |
| 2001 | 4 | 3 |
| 2002 | 9 | 12 |
| 2003 | 2 | 6 |
| 2004 | 6 | 10 |
| 2005 | 10 | 12 |
| 2006 | 8 | 14 |
| 2007 | 4 | 25 |
| 2008 | 7 | 19 |
| 2009 | 11 | 62 |
| 2010 | 16 | 74 |
| 2011 | 19 | 46 |
| 2012 | 14 | 72 |
| 2013 | 26 | 60 |
| 2014 | 19 | 63 |
| 2015 | 27 | 83 |
| 2016 | 30 | 103 |
| 2017 | 23 | 100 |
| 2018 | 30 | 87 |
| 2019 | 28 | 93 |
| 2020 | 37 | 54 |
| 2021 | 10 | 36 |
| 2022 | 18 | 31 |
| 1: For 67 persons we found side effects affecting kidneys and liver | | |
| MTX: methotrexate; ADR: adverse drug reaction | | |

| **Table S7 -** Characteristics of spontaneous reports of MTX-related ADRs by organ affected | | | | | | |  |
| --- | --- | --- | --- | --- | --- | --- | --- |
| **Characteristics** | | | **ADR on kidneys only** | **ADR on liver only** | **ADR on kidneys and liver** | **P-value** |  |
|  |  |  | **(n=298)** | **(n=1015)** | **(n=67)** |  |  |
| Age, in years | | | (n=194) | (n=546) | (n=53) |  |  |
|  | Mean (SD) | | 65.5 (13.7) | 58.9 (13.7) | 66.7 (13.7) | <0.001 |  |
| Sex | | | (n=289) | (n=1003) | (n=67) | 0.108 |  |
| Comorbidity | | | (n=207) | (n=428) | (n=55) |  |  |
|  | Cancerous | | 23 (11.1%) | 36 (8.4%) | 6 (10.9%) | 0.480 |  |
| Comedication | | | (n=298) | (n=1015) | (n=67) |  |  |
|  | Any comedication | | 245 (82.2%) | 637 (62.8%) | 57 (85.1%) | <0.001 |  |
|  | Any NSAID^1^ | | 67 (22.5%) | 152 (15.0%) | 19 (28.4%) | <0.001 |  |
|  | Acetaminophen | | 34 (11.4%) | 80 (7.9%) | 16 (23.9%) | <0.001 |  |
|  | Metamizole | | 13 (4.4%) | 11 (1.1%) | 12 (17.9%) | <0.001 |  |
|  | Non-MTX DMARD | | 120 (40.3%) | 358 (35.3%) | 29 (43.3%) | 0.152 |  |
|  |  | bDMARD | 79 (26.5%) | 234 (23.1%) | 19 (28.4%) | 0.308 |  |
|  |  | csDMARD | 53 (17.8%) | 146 (14.4%) | 14 (20.9%) | 0.147 |  |
|  |  | tsDMARD | 9 (3.0%) | 13 (1.3%) | 1 (1.5%) | 0.094 |  |
|  | Corticosteroids | | 111 (37.2%) | 238 (23.4%) | 28 (41.8%) | <0.001 |  |
|  | Folic acid | | 88 (29.5%) | 206 (20.3%) | 27 (40.3%) | <0.001 |  |
| MTX use | | |  |  |  |  |  |
|  | Route | | (n=208) | (n=793) | (n=47) |  |  |
|  |  | Oral | 142 (68.3%) | 540 (68.1%) | 35 (74.5%) | 0.693 |  |
|  |  | Subcutaneous/Subdermal | 39 (18.8%) | 174 (21.9%) | 8 (17.0%) | 0.526 |  |
|  | Duration in months | | (n=180) | (n=740) | (n=43) |  |  |
|  |  | Mean (SD) | 46.2 (66.3) | 29.9 (51.3) | 44.6 (59.5) | <0.001 |  |
| Outcome | | | (n=298) | (n=975) | (n=67) |  |  |
|  | Fatal | | 56 (18.8%) | 39 (4.0%) | 21 (31.3%) | <0.001 |  |
| 1: according to ATC classification | | | | | | |  |
| MTX: methotrexate; ADR: adverse drug reaction; NSAID: non-steroidal anti-inflammatory drug; DMARD: disease-modifying anti-rheumatic drug; bDMARD: biological DMARD; csDMARD: conventional synthetic DMARD; tsDMARD: targeted synthetic DMARD  P-values were derived using ANOVA and Fisher’s exact test | | | | | | |  |
|  |  |  |  |  |  |  |  |
